# Eosinophil Inflammation and Hyperresponsiveness in the Airways as Phenotypes of COPD, and Usefulness of Inhaled Glucocorticosteroids

**DOI:** 10.3389/fphar.2019.00765

**Published:** 2019-07-25

**Authors:** Hiroaki Kume, Masayuki Hojo, Naozumi Hashimoto

**Affiliations:** ^1^Department of Respiratory Medicine, Rinku General Medical Center, Izumisano, Japan; ^2^Department of Respiratory Medicine and Allergology, Faculty of Medicine, Kindai University, Osakasayama, Japan; ^3^Division of Respiratory Medicine, National Center for Global Health and Medicine, Tokyo, Japan; ^4^Department of Respiratory Medicine, Nagoya University Graduate School of Medicine, Nagoya, Japan

**Keywords:** airway eosinophil inflammation, bronchial hyperreactivity, asthma-COPD overlap, LABA, sputum examination

## Abstract

**Background:** The differential diagnosis in persistent airway limitation is sometimes not so clear in older adults. Airway eosinophilia and airway hyperresponsiveness may develop in some cases with chronic obstructive lung disease (COPD), independent of asthma. However, little is known about clinical significance of these phenotypes of COPD in detail.

**Aims and objectives:** This clinical study was designed to examine prevalence of airway eosinophilia and airway hyperresponsiveness in COPD who have no symptom and no past history of asthma, and to examine involvement of these pathophysiological features of asthma in the management and therapy for COPD.

**Methods:** Sputum examination *via* qualitative and quantitative procedures was performed in stable COPD (GOLD 1–3). When sputum eosinophils were qualitatively (≥+) or quantitatively assessed (≥3%), ciclesonide (inhaled glucocorticosteroids) was added on bronchodilators. In cases with FEV_1_ ≥ 70% of predicted values, acetylcholine provocation test was examined for assessment of airway hyperresponsiveness. Therapeutic effect was evaluated using spirometry and COPD assessment test (CAT).

**Results:** Sputum eosinophils were observed in 65 (50.4%) of 129 subjects using qualitative analysis; in contrast, lower grade (>0%) and higher grade (≥3%) were observed in 15 (20.3%) and 25 (33.8%) of 74 subjects using quantitative analysis. Airway hyperresponsiveness developed in 46.9% of these subjects with sputum eosinophils. Exacerbations occurred much more frequently in lower-grade airway eosinophilia without ciclesonide than in higher-grade airway eosinophilia with ciclesonide. Airway hyperresponsiveness significantly increased frequency of exacerbations in COPD with both lower and higher grade in airway eosinophilia. Addition of ciclesonide to indacaterol markedly improved lung function (FEV_1_, IC), CAT score, and reliever use in these subjects with airway eosinophilia determined by qualitative analysis. However, ciclesonide was less effective in improving these values in subjects with airway hyperresponsiveness than in those without airway hyperresponsiveness.

**Conclusions:** Airway eosinophilia and airway hyperresponsiveness are complicated with 25–50% of COPD that have no symptom and history for asthma. These phenotypes of COPD are closely related to symptom stability and reactivity to glucocorticosteroids. These phenotypes may play key roles for advancement of the management and therapy of this disease.

## Introduction

Chronic obstructive lung disease (COPD) is characterized by persistent respiratory symptoms (dyspnea, chronic cough, or sputum production) and persistent airflow limitation due to airway and/or alveolar abnormalities (The Global Strategy for the Diagnosis, Management and Prevention of COPD, Global Initiative for Chronic Obstructive Lung Disease (GOLD), 2017). COPD has an old onset with slowly progressive symptoms associated with a poor response to inhaled therapy and with longer-term smoking; in contrast, asthma generally has an early onset with intermittent symptoms associated with a good response to inhaled therapy (The Global Strategy for the Diagnosis, Management and Prevention of COPD, Global Initiative for Chronic Obstructive Lung Disease (GOLD), 2017). The pathological findings of COPD are not consistent with those of asthma. However, it may be clinically difficult to separate accurately asthma from COPD when patients present reduced lung function because diagnostic criteria for asthma due to objective findings have not been established yet. Variable clinical features (dyspnea, wheezing), which are characteristic to patients with asthma, are sometimes observed in exacerbations of COPD. Persistent airflow limitation, which is characteristic to patients with COPD, is also observed in a subset of patients suffering from asthma for a long term. Moreover, forced expiratory volume in 1 s (FEV_1_) may reduce <70% of predicted value in elderly healthy adults, referred to as lower limit of normal (LLN) (Sin et al., 2016).

Not only COPD is difficult to separate from asthma, but COPD may also be complicated by asthma in some old patients. Airway eosinophilia is related to asthma and a subset of COPD (Gibson and Simpson, 2009; Sin et al., 2016). Moreover, neutrophil inflammation is a characteristic feature in COPD and severe asthma (Brown et al., 2009; Simpson et al., 2009; Uddin et al., 2010). Recent reports have shown the overlap of asthma and COPD (ACO), which is characterized by persistent (incompletely reversible) airflow limitation with several features usually associated with both asthma and COPD (Gibson and Simpson, 2009; Global Initiative for Asthma. Global Strategy for Asthma Management and Prevention, 2017). Some consensuses for decision of ACO have also been published based on standardized values of reversibility of FEV_1_ (200–400 ml), exhaled nitric oxide (FeNO, 35–50 ppb), and eosinophils in the peripheral blood (3–5% or 150–300 cells/ml) (Gibson and McDonald, 2015; The Global Strategy for the Diagnosis, Management and Prevention of COPD, Global Initiative for Chronic Obstructive Lung Disease (GOLD), 2017). However, there is little agreement on the most appropriate cutoff values of them. These statements are an editorial, not a guideline because these are essentially opinion based, although they are supported by a large body of evidence (Miravitlles, 2017). This lack of consensus may explain the extensive data on prevalence of ACO, which is 11–56% among patients with COPD (Vanfleteren et al., 2014) and 13–61% among patients with asthma (Wurst et al., 2016). Recently, the consensus report recommends >15% and >400 ml reversibility of FEV_1_ and ≥300 cells/ml of eosinophils as criteria for diagnosis of ACO. However, all of them have not been proven yet (almost nothing beyond opinion base). To reduce a healthcare burden by these diseases, differential diagnosis should be needed to clarify them accurately by a potential biomarker (Gao et al., 2016).

The differential diagnosis among COPD, asthma, and ACO in patients with symptoms; eosinophils in the peripheral blood; and persistent airflow limitation is not always clear in older adults. Eosinophil counts in the peripheral blood may not always be consistent with eosinophil inflammation in the airways. Although it is generally considered that eosinophil inflammation and hyperresponsiveness in the airways are the major characteristic features of asthma, these pathophysiological conditions for asthma are complicated with some patients with COPD (Tashkin et al., 1996; Fujimoto et al., 2005; Leigh et al., 2006; Zanini et al., 2015; Tkacova et al., 2016; Kolsum et al., 2017), even though these patients do not have symptoms and past history of asthma. These characteristic disorders for asthma may be latent in these phenotypes of COPD. However, they are not included in the criteria for diagnosis of AOC. Therefore, evaluation of airway eosinophilia and airway hyperresponsiveness may lead to advancement of differential diagnosis and therapy among these diseases (asthma, COPD, and ACO). However, little is currently known about the clinical significance of these characteristic disorders for asthma in patients with COPD.

It may be meaningful to classify COPD according to eosinophilia and hyperresponsiveness in the airways as phenotypes of this disease. This retrospective clinical study was designed to determine whether airway eosinophilia and airway hyperresponsiveness are involved in severity and stability in the management of patients with COPD who have not been diagnosed with asthma by medical doctors, and to determine whether addition of inhaled glucocorticosteroid (ICS) is beneficial to COPD with this characteristic pathophysiology for asthma.

## Methods

### Subjects

Patients with COPD (Global Initiative Chronic Obstructive Pulmonary Disease: GOLD stage 1–3) admitted to the outpatient clinic in our hospitals (Rinku General Medical Center, Nagoya University Hospital, and National Center for Global Health and Medicine) were enrolled in this retrospective study (**Table 1**). All subjects are more than 50 years old and are current or ex-smokers of ≥20 pack-years. Diagnosis of COPD was determined based on a post-bronchodilator forced expiratory volume in 1 s (FEV_1_)/forced volume capacity (FVC) of <70% and their smoking history. Post-bronchodilator reversibility was not an exclusion criterion. None of the patients have a past history of asthma including previous doctor-diagnosed asthma, and none have characteristic features of asthma such as dyspnea and wheezing during nighttime. Moreover, none of the patients have a history of upper respiratory diseases such as allergic rhinitis and sinusitis; they have no clinical features of upper respiratory tract rhinorrhea and nasal congestion. Sputum collection and acetylcholine provocation test were approved by the research ethics committee of Rinku General Medical Center. These examinations were carried out after the consent was obtained in writing. This clinical study was approved by the research ethics committee of Rinku General Medical Center. The need for consent about the retrospective analysis of these results was waived by the committee. However, according to the committee’s suggestion, these patients were given the right to opt out this retrospective analysis by showing the contents on homepage and posters.

**Table 1:** Baseline characteristics of subjects.

Number of patients	203
Mean age	72.7 ± 8.4
Gender (men/women)	171/32
Duration (years)	6.1 ± 3.3
Reversibility (%)	5.1 ± 2.4
COPD stage (I/II/III)	38/97/68
Emphysema (+/−)	112/91
Smoking (current/ex/non)	11/192/0
Past history of asthma (+/−)	0/203

### Sputum Collection and Analysis

Sputum samples were collected by the spontaneous act of coughing up. Sputum induction by inhalation of the normal or hypertonic saline was not performed for safety because lung function is essentially reduced in patients with COPD. Sputum eosinophils were evaluated qualitatively using cytological examination in the Department of Laboratory Medicine in our hospitals. Qualitative eosinophilia was defined as positive (+) when eosinophils existed in each of 10 high-power fields. As a quantitative analysis, these sputum samples were treated with dithiothreitol, and the suspension was filtered with gauze according to the methods of Pizzichini and co-workers (Pizzichini et al., 1996). The salivary contamination was evaluated by the percentage of squamous cells in differential cell counts excluding epithelial cell. These sputum samples were used for analysis, when the percentage of squamous cells was <20% in the dithiothreitol-treated samples (Fujimoto et al., 2005). The total count of leucocytes except for squamous cells was determined with a hemocytometer. The cell suspension was adjusted to 1.0 × 10^−6^/ml, and cytocentrifuge preparations were made. Cytospin slides were stained with Diff-Quick solution. A differential cell count was carried out on 400 nucleated nonsquamous cells, and the results were expressed as a percentage of the total nonsquamous cell count. Based on this quantitative analysis, enrolled cases were classified into three groups by percentages of eosinophils, i.e., 0% (Group A), 0% < eosinophils <3% (Group B), and ≥3% (Group C).

### Acetylcholine Provocation Test

Acetylcholine inhalation challenge was carried out according to the standard method of the Japanese Society of Allergy (Makino et al., 1984), which is a modified method reported by Hargreaves and co-workers (Hargreave et al., 1981). In limited cases with FEV_1_ ≥ 70% of predicted values, this provocation test was performed for assessment of airway hyperresponsiveness to avoid the risk of respiratory failure and false positives occurring (Brutsche et al., 2006). Lung function test (flow–volume curves) was initially performed before (baseline value) and after inhalation of saline. When values of FEV_1_ at each condition were not different from those of previous test (at least more than 90%), acetylcholine provocation test was started. Lung function test for flow–volume curves was cumulatively performed at each concentration after acetylcholine (0.039, 0.078, 0.156, 0.313, 0.625, 1.25, 2.5, 5, 10, and 20 mg/ml) was inhaled by tidal breathing for 2 min each concentration similar to the previous method (Hargreave et al., 1981; Makino et al., 1984). The provocation test was interrupted at a concentration of acetylcholine where FEV_1_ is reduced by ≥20% from its baseline value. Threshold values were expressed as a minimal concentration of acetylcholine that reduces FEV_1_ by ≥20%. When FEV_1_ is not reduced ≥20% at 20 mg/ml of acetylcholine, threshold was expressed as 20 mg/ml. Although the cumulative dose curve causing a 20% reduction in FEV_1_ (PC_20_) was not calculated, airway hyperresponsiveness was defined as threshold values of <4 mg/ml of acetylcholine to maximize the specificity of diagnose of asthma (De Meer et al., 2002; Tkacova et al., 2016).

### Study Design

**Figure 1:**
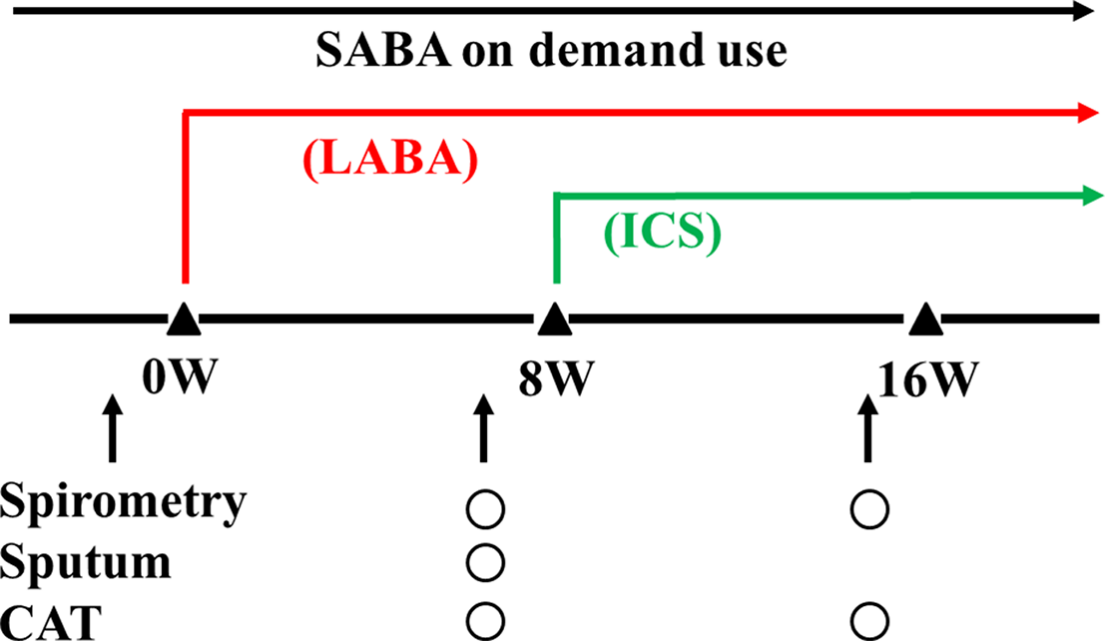
A schema for therapy and examination. Indacaterol was used as a long-acting β_2_-adrenergic receptor agonist (a LABA). Ciclesonide was used as an inhaled glucocorticosteroid (an ICS). A short-acting β_2_-adrenergic receptor agonist (a SABA) was used on demand to inhibit exacerbations.

This clinical study was carried out to determine whether eosinophilic inflammation and hyperresponsiveness in the airways are latent in patients with stable COPD and to determine whether there are differences in severity and stability in these phenotypes of COPD. Sputum examination for airway eosinophilia and acetylcholine provocation test for airway hyperresponsiveness were performed against patients with COPD diagnosed based on persistent airflow limitation (<70% of FEV_1_/FVC after inhalation of short-acting β_2_-adrenergic receptor agonists: SABA) and smoking history (≥20 pack-years). Management and therapy for COPD were carried out according to the guidelines of the Japanese Respiratory Society; on demand use of SABA and regular use of long-acting β_2_-adrenergic receptor agonists (LABA) or long-acting muscarinic receptor antagonists (LAMA) were prescribed to improve symptoms and lung function. Sputum collection was performed 8 weeks after long-acting bronchodilators were inhaled daily. ICS was added to cases with airway eosinophilia by sputum examination, which was diagnosed by qualitative (≥+) or quantitative assessment (≥3%). Inhalants (bronchodilators and glucocorticosteroids) that last for 24 h were used to reduce the number of inhalations. To assess the effects of addition of ICS on patients with airway eosinophilia and airway hyperresponsiveness, frequency of visits to the Emergency Department to improve exacerbation was investigated for 1 year in patients with COPD with sputum eosinophilia among Group A (eosinophils 0%, untreated with ICS), Group B (0% < eosinophils < 3%, untreated with ICS), and Group C (eosinophils ≥3%, treated with ICS). An exacerbation of COPD is defined as an acute event characterized by a worsening of respiratory symptoms (dyspnea, wheezing) that are beyond daily variations and lead to change in medication (GOLD). In cases where sputum eosinophils were proven using this quantitative method, tiotropium (LAMA) was administered as a bronchodilator and ciclesonide (400 μg/day) was administered as ICS. Moreover, to assess the effect of indacaterol (LABA) and additional effects of ciclesonide (ICS) to a LABA on cases of GOLD 2–3 with sputum eosinophils determined by qualitative analysis (≥+), lung function test (FEV_1_, inspiratory capacity: IC), COPD assessment test (CAT) score, and frequency of SABA for rescue use were investigated before and after regular inhalation of indacaterol once a day for 8 weeks and subsequently additional inhalation of ciclesonide once a day for an equivalent period (**Figure 1**). In this study, subjects that have elevated blood eosinophils (>300 cells/ml in peripheral blood) and reversibility in bronchodilator test (increases in >12%, > 200 ml of FEV_1_ in post-inhaled SABA) were excluded to distinguish from patients who could be judged as ACO.

### Statistical Analysis

All data are expressed as the mean ± standard deviation (SD) with 95% confidence interval (CI). Statistical significance was assessed by unpaired Student’s *t* test, one-way analysis of variance, and χ^2^ test. A probability below 0.05 (*P* < 0.05) was considered to be a significant difference.

## Results

**Figure 2:**
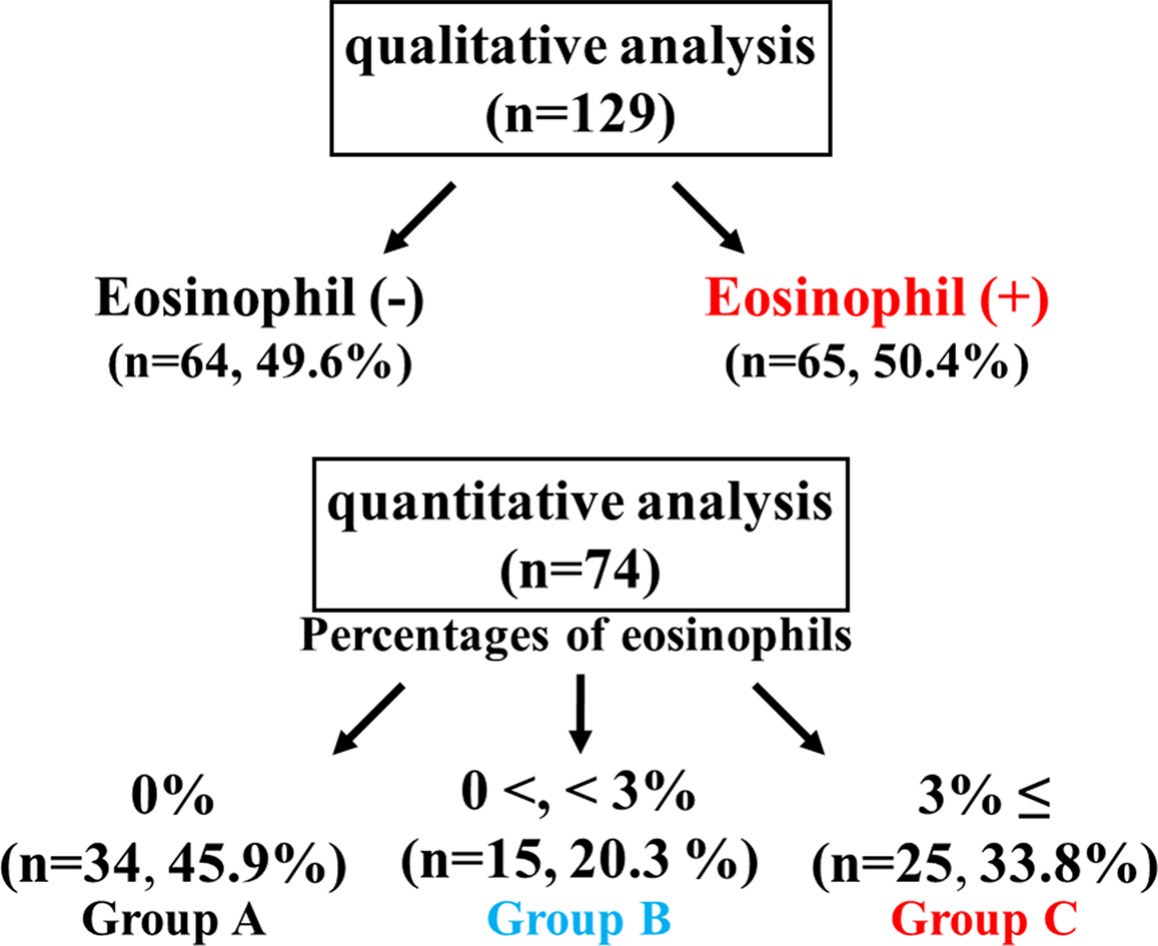
Sputum examination for airway eosinophil inflammation. Sputum eosinophilia was analyzed by quantitative or qualitative methods.

**Figure 3:**
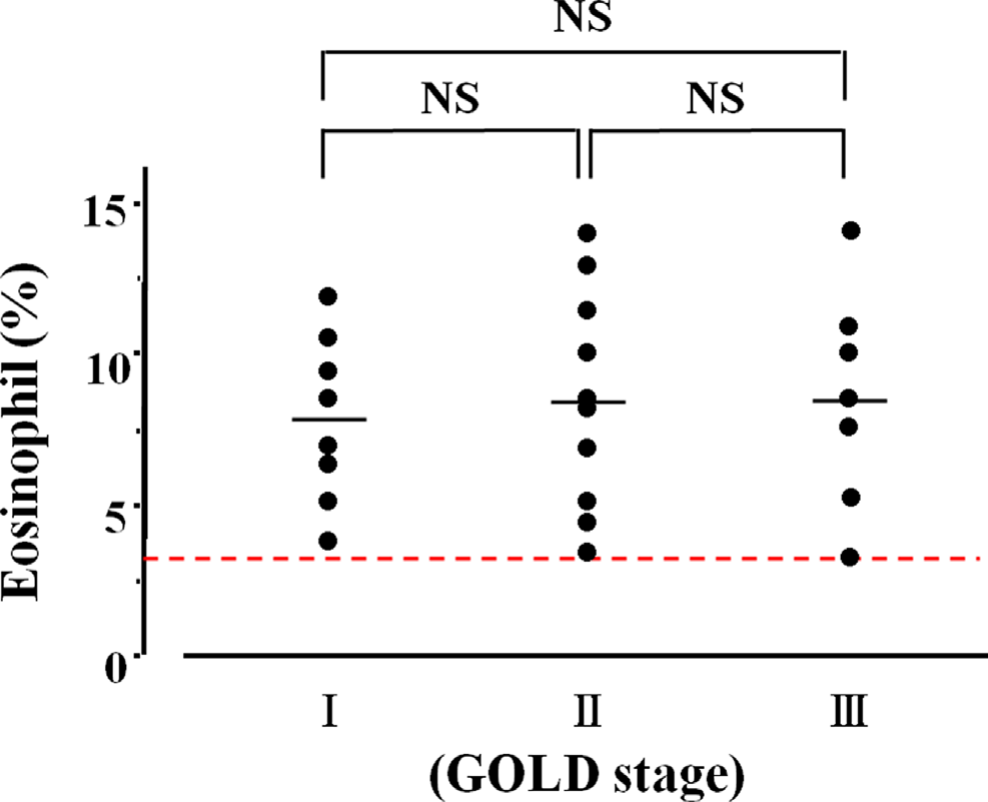
Relationship between lung function and airway eosinophil inflammation. The vertical axis is expressed as percentages of sputum eosinophils in >3%. The horizontal axis is expressed as the GOLD stage that is classified by FEV_1_. NS, not significant.

A total of 203 patients with COPD were enrolled for this clinical investigation (**Table 1**). Eosinophils in sputum were observed in 65 (50.4%) of 129 cases in the qualitative analysis (**Figure 2**). Quantitative analysis in sputum samples was carried out in 74 cases. Sputum eosinophils were not observed in 34 (45.9%) of these 74 cases (Group A). Forty (54.1%) of 74 cases had sputum eosinophils; the percentage of eosinophils was at least 3% (0% < eosinophils <3%) in 15 cases (20.3%, Group B); that percentage was ≥3% in 25 cases (33.8%, Group C) (**Figure 2**). In relation between sputum eosinophils and the GOLD stage of these 25 cases in Group C, eight cases (32.0%) belonged to Stage 1 (FEV_1_ ≥ 80% of predicted values), 10 cases (40.0%) belonged to Stage 2 (FEV_1_ ≥ 50%, < 80% of predicted values), and seven cases (28.0%) belonged to Stage 3 (FEV_1_ ≥ 30, < 50% of predicted values). The mean values of percentages of eosinophils in each stage of GOLD 1–3 were 7.9 ± 3.7 (*n* = 8) [95% CI: 4.80–11.00], 8.5 ± 3.4 (*n* = 10) [95% CI: 6.07–10.93], and 8.6 ± 3.4% (*n* = 7) [95% CI: 5.46–11.74], respectively (**Figure 3**). There was no significant relationship between sputum eosinophils and the GOLD stage (**Figure 3**).

**Figure 4:**
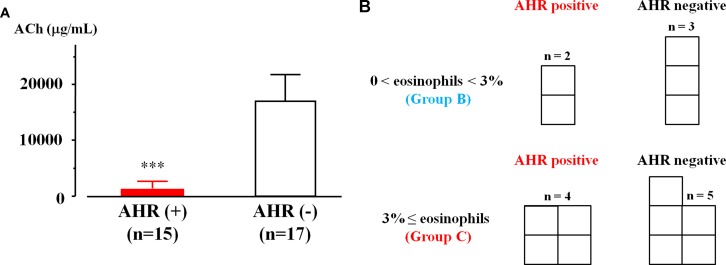
Airway hyperresponsiveness in COPD with airway eosinophilia. **(A)** Mean values of acetylcholine provocation test in sputum eosinophilis with AHR (+) and AHR (−). Sputum eosinophilis was analyzed by qualitative or quantitative procedure. **(B)** The number of patients with and without AHR in lower grade (0% < sputum eosinophils < 3%, Group B) and higher grade (sputum eosinophils ≥ 3%, Group C) of sputum eosinophilis. Sputum eosinophilia was analyzed by quantitative procedure. AHR, airway hyperresponsiveness; ACh, acetylcholine. ***P < 0.001.

Acetylcholine provocation test was performed in 32 cases that had sputum eosinophilia in qualitative or quantitative examination. In 15 (46.9%) of 32 cases, > 20% reduction in FEV_1_ was observed up to 2.5 mg/ml of acetylcholine. In contrast, in 17 (53.1%) of 32 cases, > 20% reduction in FEV_1_ was observed at ≥5 mg/ml of acetylcholine. The values of threshold in acetylcholine concentration were 1.43 ± 1.29 [95% CI: 0.69–2.18] and 16.47 ± 4.78 mg/ml [95% CI: 13.94–19.00, *P* = 0.0008], respectively (**Figure 4A**). Acetylcholine provocation test was carried out in nine cases of COPD with ≥3% of sputum eosinophils (25 cases, Group C). In four cases (44.4%), FEV_1_ was decreased by >20% up to 2.5 mg/ml of acetylcholine (**Figure 4B**). Acetylcholine provocation test was also carried out in five cases of COPD with 0% < sputum eosinophils < 3% (15 cases, Group B). In two cases (40.0%), FEV_1_ was decreased by >20% up to 2.5 mg/ml of acetylcholine (**Figure 4B**).

**Figure 5:**
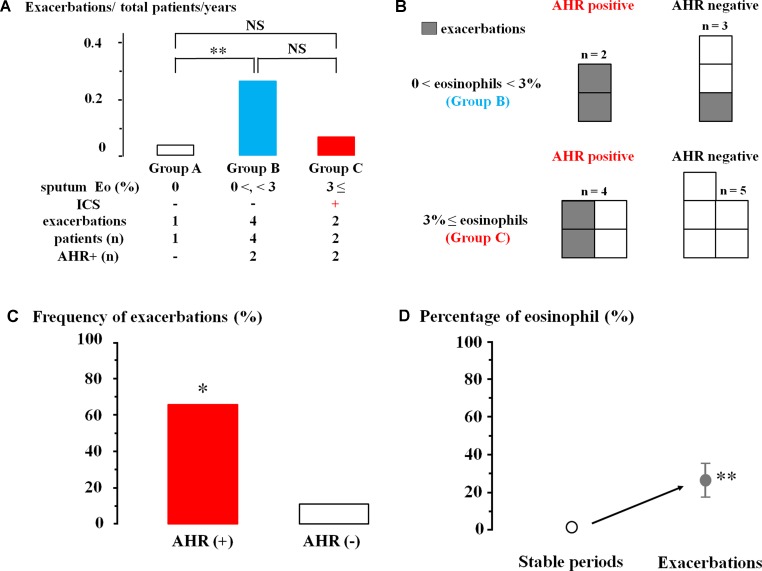
Involvement of airway eosinophilia, airway hyperresponsiveness, and ICS in occurrence of exacerbations of chronic obstructive lung disease (COPD). **(A)** Frequency of exacerbations of COPD in sputum eosinophils 0% without ICS (Group A, white column), 0% < sputum eosinophils < 3% without ICS (Group B, blue column), and sputum eosinophils ≥3% with ICS (Group C, red column). **(B)** The number of patients with exacerbations in those who underwent acetylcholine provocation test in Groups B and C. **(C)** Frequency of exacerbations in COPD with AHR and without AHR in Groups B and C. **(D)** Mean values of the percentages of sputum eosinophils in stable periods and exacerbations. AHR, airway hyperresponsiveness; ICS, inhaled glucocorticosteroid. *P < 0.05, **P < 0.01, NS, not significant.

ICS was not inhaled in 34 patients without sputum eosinophils (0% of sputum eosinophils, Group A). Exacerbation occurred in one (0.03%) of these 34 cases during follow-up for one year (**Figure 5A**). ICS was also not administered to 15 cases (0% < sputum eosinophils < 3%, Group B). Exacerbations occurred in four (26.7%) of these 15 cases (*P* = 0.009, **Figure 5A**). On the other hand, ICS was administered to these 25 patients (≥3% of sputum eosinophils, Group C). Exacerbations occurred in two (0.08%) of these 25 cases; frequency of exacerbation was not significantly different from Group A (**Figure 5A**).

In Groups B and C that have >0% of sputum eosinophils, exacerbations occurred in four (66.7%) of six cases that have airway hyperresponsiveness; in contrast, exacerbation occurred in one (12.5%) of eight cases that have no airway hyperresponsiveness (**Figures 5B, C**). Frequency of exacerbations was markedly increased in cases with airway hyperresponsiveness (*P* = 0.028, **Figure 5C**). Analysis of sputum samples collected from these four patients in the Emergency Department showed an increase of percentage of eosinophils from 1.3 ± 0.5 [CI: 0.76–1.83] to 26.5 ± 9.5% [CI: 16.53–36.47] (*n* = 4, *P* = 0.002, **Figure 5D**).

**Figure 6:**
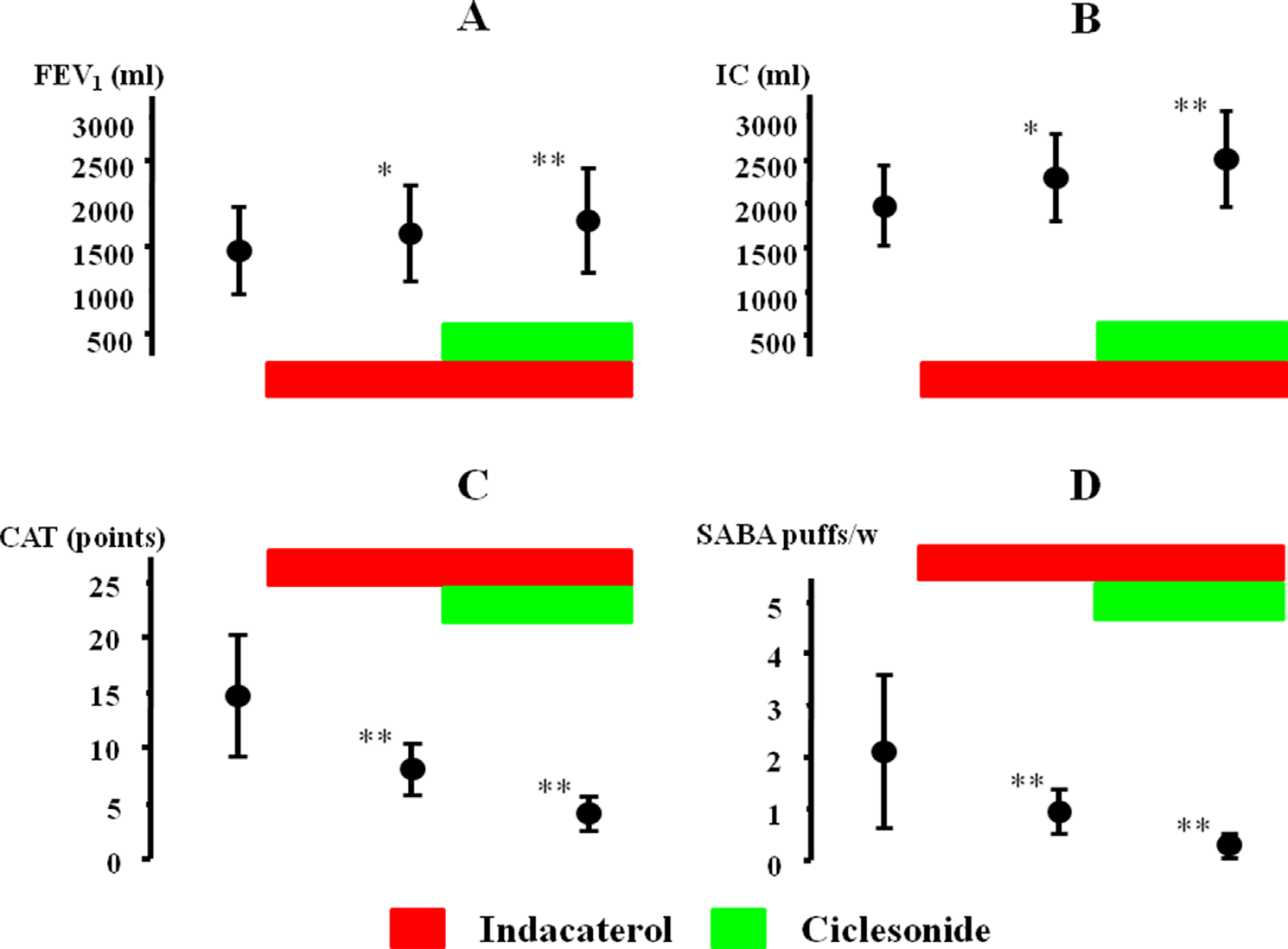
Effects of indacaterol and ciclesonide on FEV_1_ **(A)**, IC **(B)**, CAT score **(C)**, and frequency of SABA **(D)** in COPD with airway eosinophilia. *P < 0.05, **P < 0.01. IC, inspiratory capacity; CAT, COPD assessment test; SABA, short-acting β_2_-adrenergic receptor agonist.

**Figure 7:**
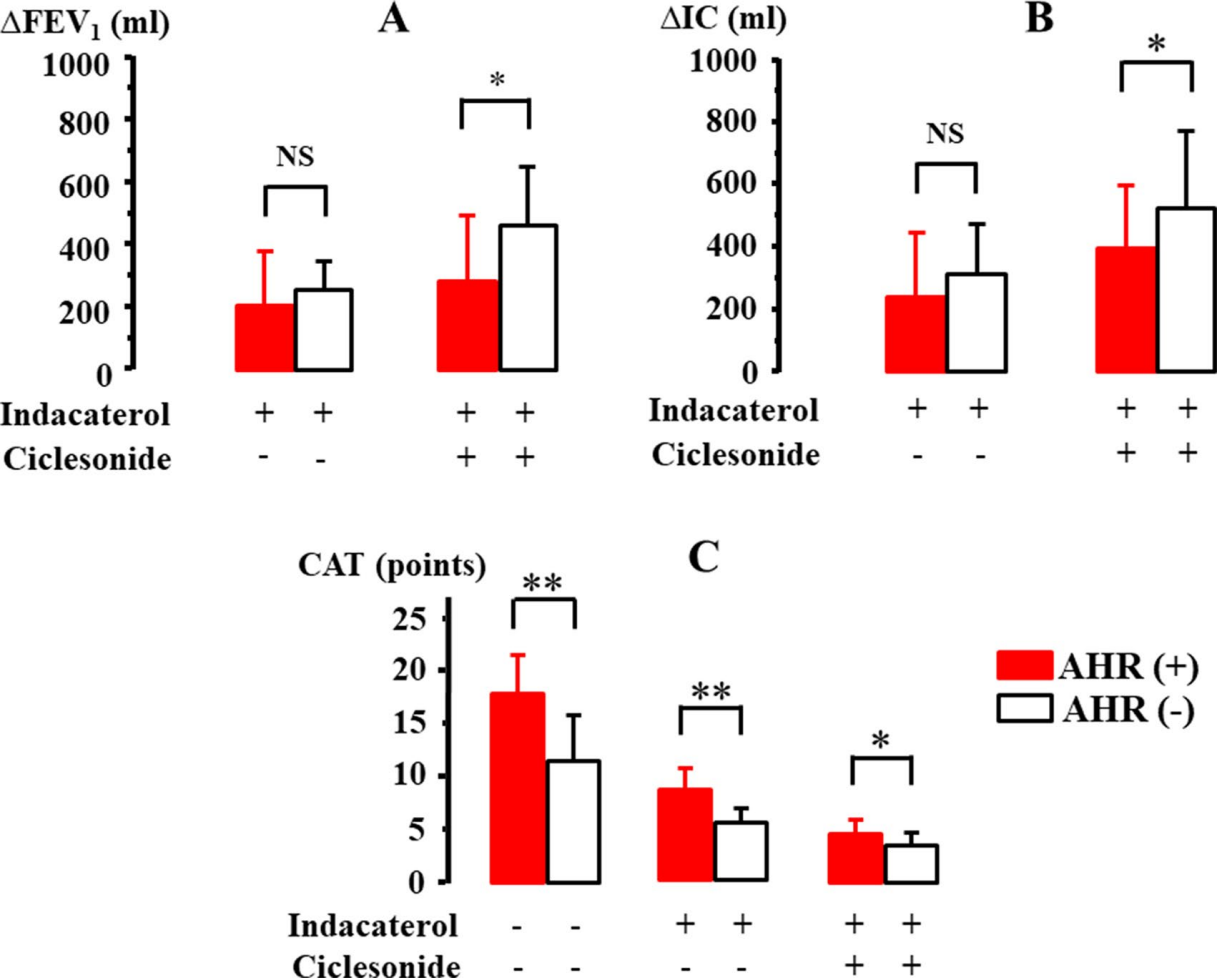
Involvement of airway hyperresponsiveness in the effect of indacaterol and cicleronide on FEV_1_**(A)**, IC **(B)**, and CAT score **(C)** in COPD with airway eosinophilia. IC, inspiratory capacity; CAT, COPD assessment test. *P < 0.05, **P < 0.01.

The effect of indacaterol and the subsequent effect of ciclesonide were investigated in 21 cases with qualitative positive airway eosinophilia (**Table 2**). After inhalation of indacaterol and indacaterol with ciclesonide, FEV_1_ was markedly augmented from 1441.9 ± 488.4 [95% CI: 1213.83–1669.97] to 1674.4 ± 534.9 [95% CI: 1424.05–1924.75] ml (Δ232.5 ml, *P* = 0.032) and to 1802.3 ± 604.7 [95% CI: 1519.29–2085.31] ml (Δ360.4 ml, *P* = 0.009), respectively (**Figure 6A**). IC was also markedly augmented from 1976.7 ± 441.9 [95% CI: 1769.88–2183.52] to 2302.3 ± 476.2 [95%CI: 2079.21–2525.17] ml (Δ325.6 ml, *P* = 0.011) and to 2523.5 ± 536.1 [95% CI: 2272.59–2774.41] ml (Δ546.8 ml, *P* = 0.006), respectively (**Figure 6B**). After inhalation of indacaterol and indacaterol with ciclesonide, CAT score was gradually decreased from 14.8 ± 5.4 [95% CI: 12.27–17.33] to 7.9 ± 2.2 [95% CI: 6.87–8.93] (*P* = 0.007) and to 4.2 ± 1.6 [95% CI: 3.45–4.95] points (*P* = 0.004), respectively (**Figure 6C**). After inhalation of indacaterol and indacaterol with ciclesonide, frequency of SABA use was also gradually decreased from 2.1 ± 1.4 [95% CI: 1.44–2.76] to 0.9 ± 0.4 [95% CI: 0.71–1.09] (*P* = 0.008) and to 0.3 ± 0.2 [95% CI: 0.21–0.39] puffs/week (*P* = 0.003), respectively (**Figure 6D**). Acetylcholine provocation test was performed in 11 cases (52.3%). FEV_1_ was reduced by >20% from baseline up to 2.5 mg/ml of acetylcholine in seven cases (mean value of threshold: 1.31 ± 0.95 mg/ml). In these cases, after administration of indacaterol and indacaterol with ciclesonide, values of FEV_1_ were increased by 208.3 ± 180.4 [95% CI: 18.91–397.69] and 283.3 ± 219.7 [95% CI: 52.66–513.99] ml, respectively (**Figure 7A**); values of IC were increased by 241.7 ± 212.3 [95% CI: 18.821–464.38] and 396.7 ± 215.7 [95% CI: 170.46–623.34] ml, respectively (**Figure 7B**). On the other hand, in four cases, FEV_1_ was not reduced by >20% even though acetylcholine was increased up to 20 mg. In these four cases, values of FEV_1_ were increased by 257.5 ± 87.9 [95% CI: 117.65–397.35, not significant] and 471.4 ± 193.7 [95% CI: 154.32–770.68, *P* = 0.034] ml, respectively (**Figure 7A**); values of IC were increased by 307.5 ± 167.73 [95% CI: 40.7–574.3, not significant] and 517.5 ± 264.0 [95% CI: 97.48–937.52, *P* = 0.024] ml, respectively (**Figure 7B**). Both FEV_1_ and IC were more increased by indacaterol with ciclesonide in cases without airway hyperresponsiveness than in cases with airway hyperresponsiveness (**Figures 7A, B**). The values of CAT score were decreased in cases with airway hyperresponsiveness from 18.0 ± 3.7 [95% CI: 14.56–21.42] to 8.8 ± 2.0 [95% CI: 6.95–10.65] and 4.8 ± 1.4 [95% CI: 3.50–6.10] after administration of indacaterol and indacaterol with ciclesonide, respectively (**Figure 7C**). Those values were also decreased in cases without airway hyperresponsiveness from 11.8 ± 4.6 [95% CI: 6.10–17.50, *P* = 0.008] to 6.3 ± 1.4 [95% CI: 4.56–8.04, *P* = 0.009] and 3.6 ± 1.2 [95% CI: 2.11–5.09, *P* = 0.046] after administration of indacaterol and indacaterol with ciclesonide, respectively (**Figure 7C**). Values of CAT score were greater in cases with airway hyperresponsiveness than in cases without airway hyperresponsiveness in each therapeutic stage (**Figure 7C**).

**Table 2:** Baseline characteristics of subjects who were inhaled on indacaterol (LABA) and ciclesonide (ICS). Sputum eosinophilia was demonstrated by the qualitative method. AHR, airway hyperresponsiveness.

Number of patients	21
Mean age	70.7 ± 5.4
Gender (men/women)	18/3
Duration (years)	0.99 ± 0.74
COPD stage (II/III)	13/8
Emphysema (+/−)	14/7
Sputum eosinophils (≥+/−)	21/0
AHR (+/−/?)	7/4/10
Smoking (current/ex/non)	0/21/0
Past history of asthma (+/−)	0/21

## Discussion

This clinical study may have demonstrated that eosinophil inflammation and hyperresponsiveness in the airways, which are characteristic features of asthma, develop in the subset of patients with COPD who do not have symptoms related to asthma and a previous diagnosis of asthma. Airway eosinophilia in COPD may have adverse effects on stabilizing symptoms but has a beneficial effect of increasing response to bronchodilators and glucocorticosteroids. Airway hyperresponsiveness in COPD may also have adversely affected stabilizing symptoms, improving lung function and quality of life. It may play a key role in the advancement of management and therapy for COPD, seeking whether these characteristic features concerning pathophysiology of asthma are hiding in patients with COPD.

Airway eosinophil inflammation was proven in stable COPD by both quantitative and qualitative analysis using sputum examination. Sputum eosinophilia was observed in 54.1% (>0%) by the former and 50.4% (≥+) by the latter. There was no significant difference between these two methods in an evaluation for sputum eosinophilia (**Figure 2**). Airway eosinophilia develops in approximately 50% of patients with COPD. The quantitative analysis is highly reliable, but that procedure is complicated. In contrast, the qualitative analysis is sufficient to seek airway eosinophilia, but not reliable for evaluation of medical intervention (the effect of inhalation therapy). Although the cutoff values of percentage of eosinophil have not been standardized yet for airway eosinophil inflammation using the quantitative methods in sputum examination, it is generally considered that ≥3% of eosinophil may be meaningful for airway eosinophil inflammation (van Veen et al., 2009), because sputum eosinophilia is less than 1% in healthy adults (Belda et al., 2000). In this study, 33.8% of the subjects had ≥3% of sputum eosinophilia (Group C), similar to a previous report (Leigh et al., 2006). To determine whether airway eosinophil inflammation has an influence on COPD, the relationship between GOLD stage (severity based on lung function) and sputum eosinophilia was examined. As shown in **Figure 3**, there was no significant difference between GOLD 1–3 and the percentage of eosinophil in sputum examination. Although little is known about an important clinical implication in <3% of sputum eosinophilia (Group B), this low-grade airway eosinophilia develops in approximately 20% of patients with stable COPD who have no clinical feature of asthma (**Figure 2**). Clinical relevance of <3% of sputum eosinophilia may be needed to investigate for advancement of management of COPD because few studies have focused on this problem.

Airway hyperresponsiveness is also a characteristic feature of asthma. Acetylcholine provocation test is generally considered to be useful and reliable for diagnosis of asthma because this clinical examination has great sensitivity and specificity to diagnose asthma (Sumino et al., 2012). On the other hand, previous reports have suggested that airway hyperresponsiveness is complicated by ∼60% or ∼94% of patients with COPD (Tashkin et al., 1992; van den Berge et al., 2012). Even though airway narrowing occurs in COPD because of airflow limitation, exclusion criteria are still not established in this provocation test for COPD. In this study, acetylcholine provocation test was carried out for subjects who have FEV_1_ ≥ 70% predicted values not only to avoid false positives but also to secure safety in this examination (Brutsche et al., 2006). Although the subjects enrolled in the study have no past history of asthma and no clinical features of asthma, airway hyperresponsiveness developed in the subset of COPD with airway eosinophilia (**Figure 4A**). Acetylcholine provocation test was carried out in 32 (30.5%) of 105 cases with sputum eosinophilia; airway hyperresponsiveness was observed in 15 (46.9%) of these 32 cases (**Figure 4A**). These results indicate that both eosinophil inflammation and hyperresponsiveness in the airways develop in at least approximately 25% in patients with COPD. It is unknown whether airway hyperresponsiveness developed in COPD is associated with airway eosinophil inflammation. However, in this study, degrees of sputum eosinophils do not relate to the complication of airway hyperresponsiveness (**Figure 4B**). Diagnosis of AOC is due to opinion base, not objective (Miravitlles, 2017). Sputum eosinophils and airway hyperresponsiveness are not included in the decision on ACO, although these pathological states are fundamental for asthma. In this study, these subjects with eosinophilia and hyperresponsiveness in the airways do not have symptoms concerning asthma and do not have ≥300 cells/ml of eosinophils in the peripheral blood and strong reversibility by bronchodilator test (FEV_1_ > 15% and >400 ml) concerning ACO; however, asthma may be latent in these patients with COPD.

To determine the role of airway eosinophilia and airway hyperresponsiveness in the management of COPD, frequency of exacerbations of COPD was examined for a year. ICS was daily administered to these patients with ≥3% of sputum eosinophils (Group C). On the other hand, ICS was not administered to patients with 0% < sputum eosinophils < 3% (Group B). ICS was not administered to patients with 0% of sputum eosinophils (Group A). Exacerbations occurred much more frequently in Group B (lower-grade airway eosinophilia without ICS) than in Group A (no airway eosinophilia without ICS) (**Figure 5A**). However, frequency of exacerbations that occurred in Group C (higher-grade airway eosinophilia with ICS) was not markedly increased compared with that in Group A (**Figure 5A**). These results indicate that airway eosinophilia causes exacerbations in COPD and that addition of ICS may be effective for reducing exacerbations in COPD with airway eosinophilia. Moreover, even though sputum eosinophil counts are <3%, anti-inflammatory therapy may be needed to make clinical course better because exacerbations occur when airway eosinophilia worsens (**Figures 4A, D**) (Papi et al., 2006; Bafadhel et al., 2011). Therefore, quantitative <3% of sputum eosinophils may be clinically meaningful for airway eosinophil inflammation, similar to the ≥3% of them. A management strategy to inhibit airway eosinophilia is beneficial to a reduction in exacerbations in COPD (Siva et al., 2007). Airway hyperresponsiveness is significantly complicated with these cases that had exacerbations in Groups B and C (COPD with >0% of sputum eosinophils) (**Figures 4B, C**). The number of exacerbations in patients with COPD is lower in Japan than in other countries (Landis et al., 2014). Since few cases also had exacerbation of COPD in this study, it may be insufficient to evaluate these results reliably. However, eosinophilia and airway hyperresponsiveness in the airways may be involved in an occurrence of an exacerbation of COPD (Zanini et al., 2015); on the other hand, the severity of airway eosinophilia and airway hyperresponsiveness in stable COPD is not involved in this phenomenon.

To investigate whether eosinophilia and hyperresponsiveness in the airways influence the management of COPD, indacaterol (LABA) and ciclesonide (ICS) were inhaled to COPD with sputum eosinophils determined by qualitative analysis (**Figure 1**). Previous reports have demonstrated that glucocorticosteroids are effective to COPD with airway eosinophilis (Pizzichini et al., 1996; Brightling et al., 2005; Leigh et al., 2006). These agents were sequentially administered to examine the effect of each agent on asymptomatic COPD with airway eosinophilia. Since indacaterol is a strong partial β_2_-adrenergic agonist, the effects of this agonist are not so markedly reduced under the conditions the number and function of these receptors are attenuated by aging (Kume, 2015; Kume et al., 2015; Kume et al., 2018). In this study, indacaterol caused a greater increase in FEV_1_ (Donohue et al., 2017) and IC (Rossi et al., 2012) than those shown in other previous reports (**Figures 6A, B**). Addition of ciclesonide to indacaterol caused much higher increases in FEV_1_ and IC (**Figures 6A, B**), and values of CAT score and frequency of SABA were markedly reduced (**Figures 6C, D**). These results indicate that ICS is effective in improving lung function, symptoms, and quality of life in COPD with airway eosinophilia (Scherr et al., 2012). Since airway inflammation induced by neutrophils and oxidative stress may be the main pathogenesis of COPD, ICS is generally considered to be not so beneficial to this disease, inconsistent with the results shown in this study (**Figures 6** and **7**). Synergistic action is generated between LABA and LAMA (Kume et al., 2015; Fukunaga et al., 2016; Calzetta et al., 2018; Kume et al., 2018); in contrast, little is currently known about additive–synergistic action between LABA and ICS. This phenomenon may due to the effectiveness of ICS on airway eosinophil inflammation in COPD, similar to asthma (van Rensen et al., 1999). Next, in the investigation for effects of airway hyperresponsiveness on COPD with airway eosinophilia, there was no deference in response to indacaterol for FEV_1_ and IC between these subjects with and without airway hyperresponsiveness; in contrast, ciclesonide caused greater increases in FEV_1_ and IC in cases without airway hyperresponsiveness than in cases with airway hyperresponsiveness (**Figures 7A, B**). Since indacaterol has higher intrinsic efficacy, that effect may not be attenuated against increased reactivity to muscarinic action (airway hyperresponsiveness) (Kume and Ito, 2017; Kume, 2018). In contrast, although glucocorticosteroids have no direct effect on muscarinic contraction of airway smooth muscle, these agents may improve airway hyperresponsiveness in asthma (Bel et al., 1991; Currie et al., 2003). However, mechanisms underlining this reduced responsiveness to glucocorticosteroids in COPD with airway hyperresponsiveness have not been investigated in detail. CAT sore was gradually decreased by these agents; however, that value was greater in the subjects with airway hyperresponsiveness than in those without airway hyperresponsiveness at each phase of treatment (**Figure 7C**). Although quality of life is improved by ICS with LABA in COPD (Scherr et al., 2012), increased reactivity to muscarinic action may lead to make quality of life in patients with COPD worse, similar to asthma (Porsbjerg et al., 2007; Scherr et al., 2012); however, the reason for this result is unknown. Limitations of this study include the fact that not many patients were able to undergo acetylcholine provocation test because of their airflow limitation and that few patients caused an exacerbation. Since time since COPD diagnosis is 8.0 (Kolsum et al., 2017) and 6.1 years (Kostikas et al., 2018) in other clinical trials, mean duration of this disease in total patients of this study was similar to values shown in a previous report (**Table 1**). However, mean duration of COPD in 21 cases was relatively short compared to such values, as shown in **Table 2**. One reason for this is that patients who have never been diagnosed with COPD and treated using ICS and bronchodilators before coming to our hospitals were fundamentally enrolled in this study. In this study, the disproportional gender imbalance was also observed, as shown in **Table 1**, compared to other reports (Leigh et al., 2006; Tkacova et al., 2016; Kolsum et al., 2017). A possible reason considered is that the smoking rate in women is much less than that in men in the older Japanese population (≥60 years old) (Fujimoto et al., 2005).

**Figure 8:**
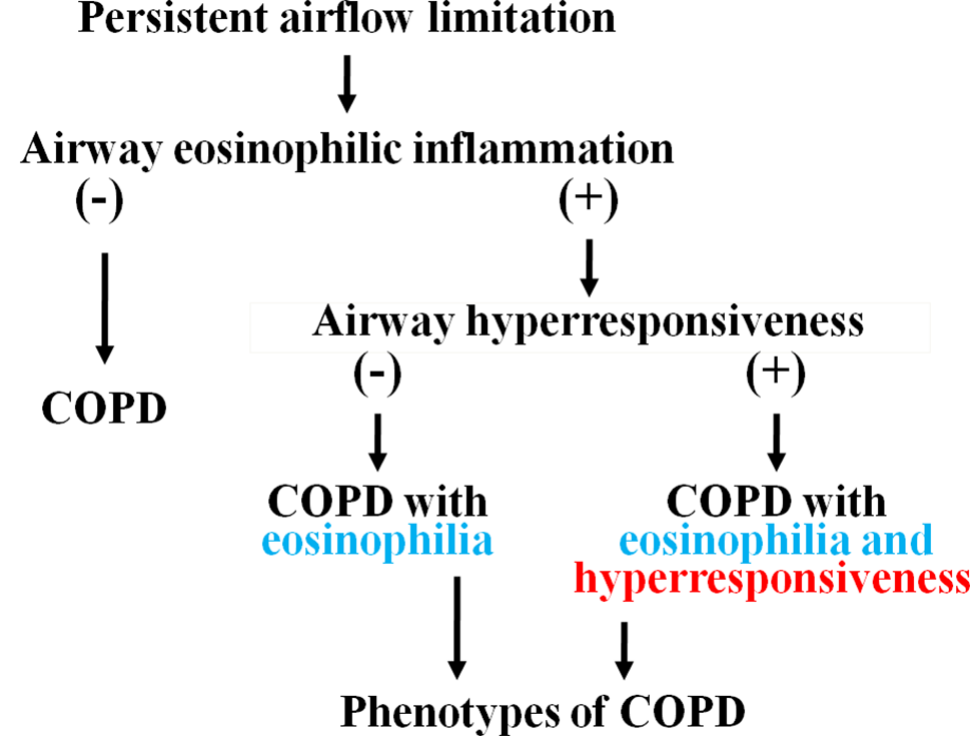
Phenotypes of COPD classified into airway eosinophilia and airway hyperresponsiveness.

In conclusion, airway eosinophil inflammation develops in approximately 50% of patients with COPD; moreover, airway hyperresponsiveness develops in approximately 50% of patients with airway eosinophilia (approximately 25% of total patients with COPD) (**Figure 8**) (Zanini et al., 2015). Mechanisms of this phenomenon have not been elucidated; however, eosinophils as well as neutrophils may be involved in the pathophysiology. Asthma may be latent in the latter cases since these cases have fundamental pathophysiology of asthma, although these cases have no typical symptoms for asthma and bronchial reversibility, which are included in evaluation of ACO. Stability and response to ICS in these phenotypes (airway eosinophilia and airway hyperresponsiveness) of COPD are different in COPD without eosinophilia and hyperresponsiveness in the airways. Therefore, investigation of these phenotypes may lead to advancement of the management and therapy for COPD.

## Author Contributions

HK, MH, and NH participated in research design. HK contributed to case registration. HK, MH, and NH discussed data analysis.

## Conflict of Interest Statement

The authors declare that the research was conducted in the absence of any commercial or financial relationships that could be construed as a potential conflict of interest.
